# Implementation of C-Reactive Protein Point-of-Care Testing for Respiratory Tract Infections in Primary Care: A Systematic Review Using the RE-AIM Framework

**DOI:** 10.3390/antibiotics15090848

**Published:** 2026-08-31

**Authors:** Natalie J. Moss, Stephanie A. Marsh, Colin H. Cortie, Mary A. Burns, Andrew Bonney, Judy Mullan

**Affiliations:** Graduate School of Medicine, Faculty of Science, Medicine and Health, University of Wollongong, Wollongong, NSW 2518, Australia; smarsh@uow.edu.au (S.A.M.); marybu@uow.edu.au (M.A.B.); abonney@uow.edu.au (A.B.); jmullan@uow.edu.au (J.M.)

**Keywords:** CRP POCT, primary care, RE-AIM, systematic review, respiratory tract infection, implementation science

## Abstract

Background/Objectives: C-reactive protein point-of-care tests (CRP POCTs) have become a useful tool in reducing inappropriate prescribing of antibiotics in the management of respiratory tract infections (RTIs). This review examined the barriers and enablers of CRP POCT implementation in primary care using the Reach, Effectiveness, Adoption, Implementation and Maintenance (RE-AIM) framework. Methods: A search was conducted in six electronic databases (PubMed, Scopus, CINAHL (EBSCOhost), Medline (EBSCOhost), ProQuest and CENTRAL) for studies up to December 2025 on CRP POCT implementation for RTIs in primary care. Eligible studies included quantitative randomised controlled trials (RCTs), qualitative studies, cost-effectiveness studies, mixed-methods and observational studies. Only English-language studies were included. Results: The review included 35 studies. CRP POCT was generally found to be an effective and acceptable tool due to reductions in antibiotic prescribing, playing a role in supporting clinical decision-making and patient communication. However, widespread adoption and usage were inconsistent across studies. Further, qualitative findings raised operational and financial constraints as key barriers to sustained implementation. Reporting of RE-AIM domains varied across study type. Notable gaps were identified in reporting of the maintenance domain along with population representativeness, non-participation, and device uptake. Of the 35 studies, 13 included ‘operational’ and/or ‘financial constraints’ as identified themes or concerns about wider implementation. Conclusions: Although CRP POCT has routinely been found to be effective, a wider comprehensive assessment of implementation is restricted due to gaps in reporting. Key barriers to sustained and scalable global implementation of CRP POCT in primary care are predominantly financial and operational.

## 1. Introduction

Antimicrobial resistance (AMR) is ranked by the World Health Organisation as one of the top 10 threats to global health [1]. Global predictions estimate that over 8 million deaths annually can be attributable to AMR, with projections suggesting an additional 169 million deaths by 2050 if unaddressed [2]. Without further effective interventions, AMR has been forecast to place a significant financial burden on health systems worldwide [3].

Antibiotic usage is a key driver of AMR, with strong evidence linking increased use to rising resistance [4,5]. This burden is exacerbated by the inappropriate use or unnecessary prescribing of antibiotics, particularly in primary care settings [6,7]. Prescribing for acute respiratory tract infections (RTIs) represents one of the largest contributors to inappropriate antibiotic prescribing, as many RTIs are viral in origin and do not require antimicrobial therapy [8,9]. Recent global estimates indicate that approximately 45% of antibiotics prescribed for RTIs are inappropriate, highlighting an important target for interventions [10]. For example, community prescribing rates for RTIs in Australia have been reported as being four to nine times higher than recommended clinical guidelines [8].

In response to rising AMR, antimicrobial stewardship (AMS) programs have grown in prevalence around the world. These programs promote responsible antimicrobial use to preserve antimicrobial effectiveness, minimise harm and reduce the emergence of AMR [11,12]. AMS strategies are multifaceted, ranging from schemes targeting governance and policies to more specific interventions that address key areas of focus [11,12]. In primary care, where most antibiotics are prescribed, there is a need to focus on interventions that influence clinicians’ prescribing behaviours and improve patient communication and education [13].

One promising intervention for improving the management and treatment of RTIs is C-reactive protein (CRP) point-of-care tests (POCT). CRP POCT has emerged as a simple and useful clinical decision support tool to aid diagnosis and prescribing in clinical practice. CRP POCT has demonstrated efficacy in supporting appropriate antibiotic prescribing at the index consultation with an average reported 21–24% reduction in inappropriate prescriptions [14,15]. Further, CRP POCT thresholds have become increasingly recognised and integrated into clinical guidelines for the management of RTIs in Europe [16,17]. However, the adoption of CRP POCT globally varies widely [18]. While uptake in primary care is as high as 50% for RTI consults in Norway [19] and 45% in Denmark [20], implementation elsewhere remains limited. Despite availability outside parts of Europe, the use of CRP POCT in primary care is still constrained [19,20]. Despite strong evidence of effectiveness in reducing prescribing rates and successful uptake in select settings, the broader implementation of CRP POCT remains inconsistent, highlighting the need to better understand the factors influencing its implementation into routine care.

This systematic review aimed to assess the implementation of CRP POCT in primary care using the Reach, Effectiveness, Adoption, Implementation, and Maintenance (RE-AIM) framework. RE-AIM is a widely used evaluation and planning framework for assessing implementation success [21], and was selected for this review due to its demonstrated utility in evaluating implementation outcomes across diverse settings and heterogeneous study designs [22,23]. While the effectiveness of CRP POCT in reducing antibiotic prescriptions for acute RTIs in primary care has been well established [14,15], to the authors’ knowledge, this is the first review to apply the widely used RE-AIM framework to evaluate its implementation in this setting. This is important because the benefits of CRP POCT can only be realised when the intervention is successfully adopted, implemented and sustained in routine clinical practice. This review examines the barriers and enablers to implementation across the Reach, Effectiveness, Adoption, Implementation and Maintenance (RE-AIM) dimensions.

## 2. Results

### 2.1. Summary

**Figure 1:**
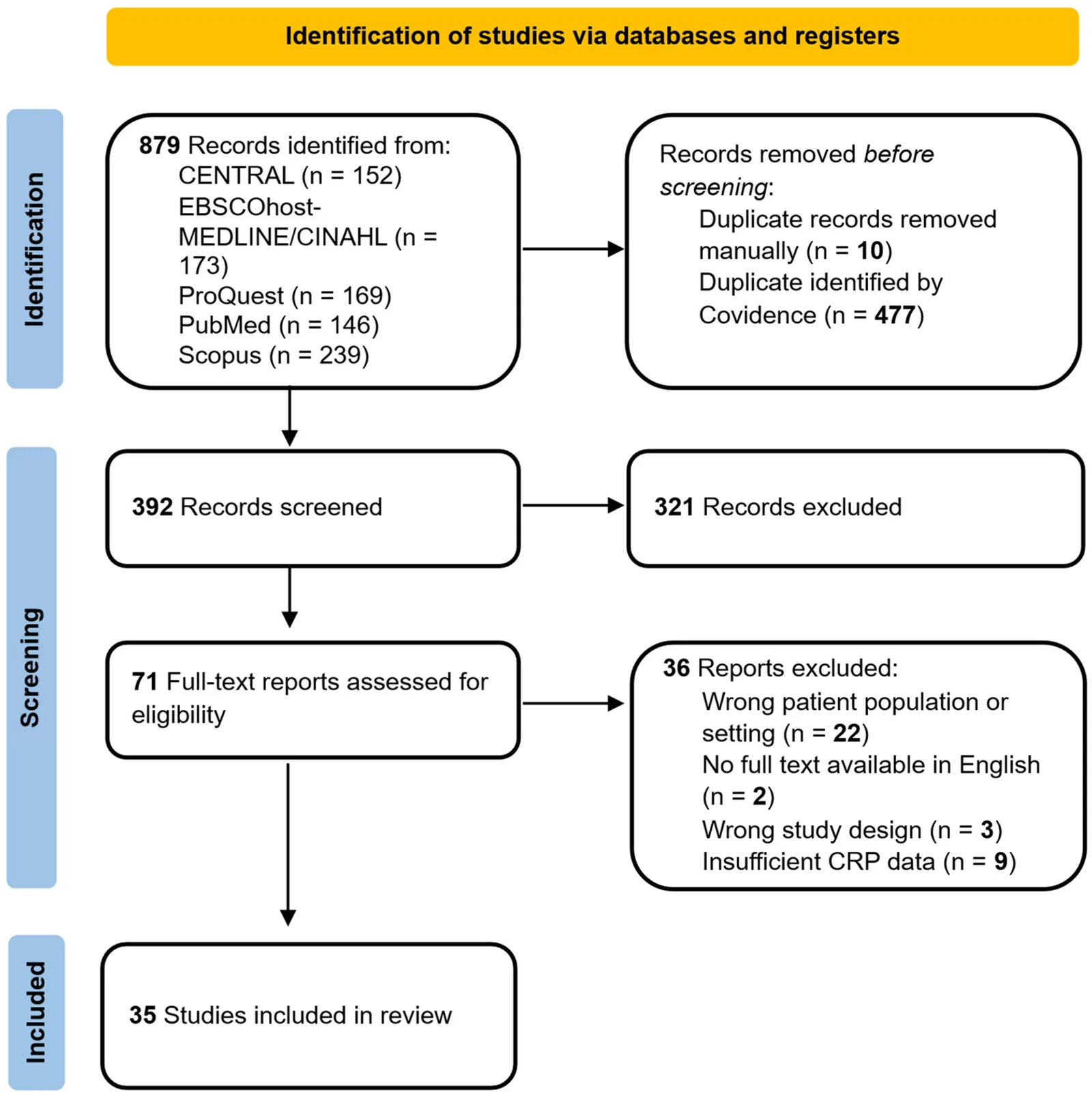
PRISMA 2020 flow diagram outlining the screening and study selection process.

The database search initially identified a total of 879 studies across six databases. Following the removal of duplicates and screening of abstracts and titles, 71 studies met the inclusion criteria and were included for full-text review. Of these, 36 studies were excluded by consensus of three researchers (N.M., S.M., J.M.) with reasons listed in Figure 1. This resulted in a total of 35 publications included in this review, of which 26 were treated as individual studies, as several trials had multiple publications or reported on different study components. The study selection process is summarised in the PRISMA 2020 flow diagram (Figure 1).

### 2.2. Study Characteristics

Of the 35 studies included in this review, ten were RCTs [22,23,24,25,26,27,28,29,30,31], ten were qualitative studies [32,33,34,35,36,37,38,39,40,41], seven were economic or cost analyses [42,43,44,45,46,47,48], and eight were classified as other [49,50,51,52,53,54,55,56]. The ‘other’ category included mixed-methods or observational studies. A summary of key study information is outlined in Table 1. Most of the studies included in the review were conducted in Europe (31/35, 88%) and the Afinion^TM^ CRP POCT device (Abbott Technologies, Los Angeles, CA, USA) was the most frequently examined device (26%, 9/35). In contrast, 14% (5/35) of studies did not directly use a device in their analysis, and an additional 11% (4/35) did not state the CRP POCT device used.

### 2.3. Reach

Within the Reach domain, which assesses target populations and representativeness, considerable heterogeneity was observed due to the inclusion of diverse study types. Of the 35 studies, 12 (34%) focused solely on patient participants [22,23,24,25,26,27,28,29,30,31,49,52], 11 (31%) focused on clinicians and other healthcare professionals as participants [32,33,34,35,36,37,38,39,53,55], and five (14%) included both patients and clinicians as participants [40,41,51,54,56]. Of the studies with adult patients as their populations, seven examined patients presenting with lower RTIs with acute coughs [22,23,24,26,27,30,49], three (9%) examined adult patients presenting with upper RTIs [28,29], with one also including rhinovirus infections [25].

Although all studies reported on participant populations and sample sizes, reporting of recruitment processes and non-participation was limited. Among the few studies that documented sites that declined participation, time constraints and availability issues were identified as key reasons for non-participation among clinicians and practices [23,30,37]. Across the study designs, RCTs provided the most comprehensive reporting of the Reach domain, enabling clearer assessment of the intended target population and who ultimately participated. In contrast, economic evaluation studies provided the least detailed reporting on populations, recruitment, and exclusions, often relying on hypothetical or modelled populations derived from the existing literature [43,44,46]. This inconsistency resulted in limited reporting transparency.

Additionally, the assessment of representativeness, a core component of the Reach domain, was limited across all study types due to incomplete reporting and the lack of comparisons between target populations and observed study samples. Outside of age and sex, which were reported in most studies, reported participant characteristics varied considerably across studies. Three studies (9%) recorded rural/metropolitan locale information for patients [38,39,51], while nine studies (26%) included education status/practice experience [23,25,31,32,36,38,39,50,55]. Patient participant ethnicity was only reported in one study [40], and socioeconomic status was mentioned in another study [30]. Thus, this heterogeneity in reporting limited the ability to assess the representativeness of study populations.

### 2.4. Effectiveness

Within the Effectiveness domain, which refers to clinical effectiveness, the primary outcomes reported across most quantitative studies (excluding qualitative designs) were the impact of using CRP POCT on the reduction in antibiotic prescribing and the viability of its use in clinical decision-making and practice. Most of the RCTs (9/35, 26%) found some reduction in antibiotic prescribing associated with using CRP POCT in clinical practice, though the significance and degree of reduction varied, ranging from 6.3 to 22% in initial studies [22,23,25,26,27,28,30] and 1.1 to 5% in follow-up studies 12 months or longer [24,29]. Notably, several studies reported an enhanced reduction in prescribing when CRP POCT was combined with additional communication training [23,28,30].

Assessment of potential adverse events or negative outcomes is an important component of the Effectiveness domain but was relatively limited across the included studies. Except for one mixed-methods study [56], six RCTs (17%) [23,24,25,26,27,28] reported on safety-related measures such as adverse events and patient recovery outcomes; however, these measures were not consistently evaluated across studies. Among the non-randomised study designs included in this review, CRP POCT was found to influence clinical decision-making and prescribing practices to varying degrees [50,51,52,53]. Clinicians in three studies (9%) reported satisfaction with CRP POCTs and perceived them as a useful adjunct to clinical assessment [51,54,56]. Findings from the qualitative studies also echoed these outcomes, with clinicians commonly linking perceived usefulness to increased diagnostic confidence and improved patient communication [32,33,34,35,36,37,38,40,41]. However, despite these generally positive findings, some studies identified reluctance towards CRP POCT among some clinicians [37,53], with one study stating that these views were predominantly raised by clinicians who had extensive experience in practice [35].

### 2.5. Adoption

The adoption domain considered device usage, acceptability, and engagement during trials along with examining any common variation in protocols and compliance. Uptake of CRP POCT across and within studies varied considerably, reflecting differences in approaches to implementation, training, and device usage. Only four studies (11%) mandated and measured CRP POCT use in all eligible consultations [22,23,25,28]. In contrast, when use of the device was optional, utilisation rates were highly variable within and between studies. Among the 16 studies (46%) that reported device utilisation rates, three studies (9%) reported very low usage, with CRP POCT used in less than 6% of eligible consultations [24,26,29]. Utilisation across the remaining studies fluctuated from 26% to 98% [30,49,50,51,52,53,54,55], indicating substantial heterogeneity in adoption at the clinical and practice levels.

Qualitative findings provided further insight into the varied rates of engagement across studies. Some clinicians described that they did or would only use CRP POCT when faced with diagnostic uncertainty, rather than as a routine component of care [32,33,36,50]. Further, there was also inconsistent reporting on device handling, with some studies describing clinicians using the device, whilst others reported or accounted for other staff or practice nurses to be the ones utilising CRP POCT in practice [25,26,46,53].

Acceptability among patients and clinicians, an important component of Adoption, was measured through qualitative reporting for this domain. Clinicians’ views on the acceptability of the device were largely positive, with acceptability reported largely in relation to positive impacts on clinical decision-making and patient communication [32,34,35,37,38,39,40,41,53,54,56]. Although two studies (6%) described hesitations linked to perceived barriers (e.g., operational or financial concerns) [35,56], patient acceptability was more frequently positive when examined. Specifically, nine studies (26%) assessed patient perspectives and consistently reported high levels of satisfaction or mostly positive views regarding CRP POCT [23,26,34,37,38,40,41,54,56].

### 2.6. Implementation

The Implementation domain primarily assessed cost, fidelity to protocol and delivery considerations, which varied considerably across studies. The seven studies (20%) that included economic or cost-effectiveness analyses [42,43,44,45,46,47,48] assessed the cost-effectiveness of CRP POCT across eight countries in Europe and elsewhere. Despite variances in health systems, populations, and devices, studies generally identified CRP POCT to be cost-effective when considered in the context of broader healthcare settings and efforts to mitigate growing AMR or healthcare costs. However, across varying settings, these studies still indicated additional costs compared to standard care, with limited willingness to pay thresholds. Further, two studies (6%) identified adherence to CRP POCT prescribing guidelines as a key determinant of cost-effectiveness, highlighting the importance of implementation fidelity [45,47].

Adherence (fidelity) in the application of CRP POCT for RTI management was also measured as part of the implementation domain. Protocol adherence and adaptations were inconsistently reported across the varied study types. Notably, two studies (6%) reported the use of CRP POCT beyond the management of RTIs [51,52], whilst three (9%) others identified or accounted for some degree of non-adherence to prescribing protocols and trial guidelines in their analysis [26,27,45].

Qualitative studies contributed important contextual information for the implementation process, examining perceptions on workflow and perceived barriers. The most commonly reported barriers related to workflow and trial implementation included operational challenges, concerns regarding device accessibility, and time constraints. Clinician perspectives on the impact of CRP POCT on workflow and consultation time duration varied across some studies [34,36,37]. However, increased consultation time was more frequently identified as a larger concern in several others [33,35,38,39,40,41,53,54]. In contrast, one study reported that patients were generally unconcerned about longer consultation times [40].

Operational factors, particularly device accessibility and placement within a clinical setting, were also frequently highlighted as important determinants of successful implementation. Variations in how devices were integrated within a practice appeared to influence the perception of impact on practice workflow [34,35,37,38,40,53,54]. Additional implementation considerations, including device maintenance, handling, and performance, were reported less frequently but contributed to perceived implementation burden in some studies [37,41,56].

### 2.7. Maintenance

The Maintenance domain predominantly assessed the long-term sustainability of CRP POCT by examining follow-up periods of more than 6 months and qualitatively exploring participants’ perspectives and reasoning about its continued use. Maintenance was the least frequently reported RE-AIM domain, with only two studies (6%) [24,29] including follow-up periods of 6 months or longer, both of which were extensions of larger primary trials [23,28]. Both follow-up studies, one conducted at 12 months [29] and the other 3.5 years [24], reported notably low or declining usage rates of CRP POCT, with utilisation observed in only 3.7% and 5.77% of eligible RTI consultations, respectively. Correspondingly, both studies noted an increase in antibiotic prescribing rates within the CRP POCT intervention groups at these time points, compared with their original trial findings.

Qualitative findings related to the maintenance domain included clinicians’ and patients’ perceptions of their willingness to use CRP POCT in future consultations, as well as identified barriers and facilitators to sustained use. Ten studies (29%) provided insight into potential long-term adoption by examining patient and/or clinician willingness to use the devices in the future [26,32,33,34,35,36,38,40,41,56]. Patient willingness was only described in two studies (6%), with almost all patients reporting being happy with the future use of CRP POCT [26,41]. In contrast, clinicians’ willingness to adopt CRP POCT, when assessed, was more variable. While several studies reported high levels of clinician acceptance [34,38,41,56], others highlighted more mixed or cautious perspectives [32,33,35,36,40].

Reported willingness to sustain long-term CRP POCT use was closely associated with perceived barriers and facilitators. The most common barriers included operational challenges (e.g., workflow integration and device accessibility) [32,35,37,38,39,40,41,53,54,56] and financial concerns, such as costs and reimbursement [34,35,36,37,39,54]. A smaller number of studies also identified clinician concerns regarding the accuracy of the device [26,36,41] and over-reliance on the test [34,36,41]. Additionally, although less frequently reported, key facilitators in the sustained use of CRP POCT included the provision for adequate training and financial or organisational incentives to support continued use [33,37,53,54].

## 3. Discussion

This review applied the RE-AIM framework to evaluate the implementation of CRP POCT for the management of RTIs in primary care, highlighting several important findings and gaps in the literature. Overall, CRP POCT was viewed positively by clinicians due to its role in reducing antibiotic prescribing, supporting clinical decision-making and facilitating patient communication. However, widespread adoption and usage remained inconsistent, with operational and financial challenges emerging as the most commonly reported or perceived barriers. In addition, variability in reporting across studies, particularly regarding participant representativeness, recruitment processes, usage rates, and long-term outcomes, limited a comprehensive assessment of implementation.

Within the Reach domain, most studies reported basic participant characteristics, but important variables such as ethnicity, geographic distribution, and socioeconomic status were often inconsistently reported or were absent. Assessment of representativeness was therefore limited by incomplete demographic reporting and a lack of information on the target populations represented by the study samples. Similarly, recruitment processes and reasons for non-participation were rarely described, restricting understanding of factors influencing clinician or practice engagement. These reporting limitations reflect broader challenges within implementation research, where indicators of representativeness and non-participation are frequently not reported [58,59].

The Effectiveness domain was generally well described, with most studies reporting reductions in antibiotic prescribing, improvements in clinical decision-making, and positive perceptions among clinicians. However, the magnitude and significance of reductions in prescribing varied. Studies combining CRP POCT with communication training reported greater reductions in prescribing [23,28,30], highlighting the value of complementary interventions. Variability in outcomes may partly reflect inconsistent use of CRP POCT, as several studies reporting non-significant or smaller effects also documented low utilisation rates [24,26,29,53]. For instance, both Eley et al. [26] and Do et al. [60], examining both adult and pediatric populations, have attributed limited reductions in antibiotic prescribing to low uptake of CRP POCT. Although qualitative data provided some insight into the clinical decisions to use the test, the reasons underlying low uptake were rarely explored, limiting understanding of how implementation strategies could be optimised.

The Adoption and Implementation domains highlighted persistent operational challenges affecting routine use. While clinicians generally accept the clinical value of CRP POCT, concerns about workflow integration, resource requirements, and responsibility for conducting tests remain common. Perceptions of the impact on workflow varied according to who was responsible for performing tests, with responsibility appearing to fall primarily on clinicians or practices, alongside variability in protocols, adherence and integration into routine care [34,35,37,38,40,53,54]. However, resource management and operational concerns surrounding POCT have additionally been raised as areas of interest in the wider literature [61,62], highlighting the importance of addressing the feasibility of these devices in practice. Device-related factors may also contribute to operational challenges. Many CRP POCT devices require ongoing maintenance and replacement of cassettes and reagents, which may limit accessibility in practice [63,64,65]. Smaller single-use devices stored within consultation rooms may help overcome barriers, although further research is needed to evaluate and make comparisons about their impact on workflow and consultations. The Maintenance domain was the least reported, highlighting an important evidence gap relating to long-term sustainability. Only two follow-up RCTs investigated long-term outcomes, both reporting declining CRP POCT use alongside increased antibiotic prescribing compared with their original trials, suggesting challenges in sustaining implementation fidelity over time. Evidence from qualitative studies consistently identified financial and operational demands as major barriers to ongoing use. Although economic evaluations indicate that CRP POCTs are cost-effective, reimbursement mechanisms and cost allocation remain important concerns for clinicians and healthcare organisations. International experience suggests that governance structures, financial incentives and policy frameworks are key determinants of sustained implementation and future success [19,35,37,61,62]. Further, Scandinavian countries, such as Norway and Sweden, have demonstrated successful implementation supported by coordinated policy and funding arrangements [19,20,66]. Collectively, these findings highlight the need for longitudinal implementation research and coordinated policy initiatives that facilitate sustainable adoption into routine primary care.

Several limitations of this review should be acknowledged. First, the search was limited to English-language publications and excluded ‘grey’ literature, which may have limited the breadth of our findings. Grey literature was not included due to concerns about the absence of peer review and methodological inconsistency across sources. Second, this review focused on adult populations within primary care settings, despite a growing body of literature investigating the use of CRP POCT in pediatric populations and other healthcare settings. While primary care accounts for a large proportion of antibiotic prescribing, CRP POCT is increasingly being used in emergency care and is also being evaluated in community pharmacy settings. Broadening the scope to include these settings and populations may have provided further insights into shared and context-specific implementation barriers and facilitators. Finally, most included studies were conducted in Europe, which may limit the generalisability of the findings to other regions and healthcare systems. Future research investigating the implementation of CRP POCT testing in diverse geographic contexts, particularly in low- and middle-income countries, is needed to determine whether similar implementation themes emerge. Comparative studies across different healthcare settings and populations could further strengthen understanding of implementation processes and inform more comprehensive strategies for the adoption and sustained use of CRP POCT.

## 4. Materials and Methods

### 4.1. Design

This systematic review has been pre-registered with PROSPERO, the International Prospective Register of Systematic Reviews (registration number CRD420251243866) and was conducted and reported in accordance with the Preferred Reporting Items for Systematic Reviews and Meta-Analysis (PRISMA) guidelines [67]. A change in setting from ‘Community care’ to ‘Primary care’ was registered in PROSPERO [21 August 2026].

### 4.2. Information Sources and Search Strategy

Six electronic databases (PubMed, Scopus, CINAHL (EBSCOhost), Medline (EBSCOhost), ProQuest and CENTRAL) were searched from inception on 1 December 2025. Searches combined keywords (including synonyms and word variations) and MeSH terms using Boolean operators (AND/OR). Search terms addressed three concepts: ‘Point of care testing’, ‘C-reactive protein’ and ‘respiratory tract infection’ combined using ‘AND.’ Full search strategies are provided in Table S1.

### 4.3. Eligibility Criteria

Inclusion and exclusion criteria were predefined. Studies were limited to English-language publications with ‘grey’ literature excluded. Eligible study designs included: randomised controlled trials (RCTs) or non-randomised trials (including cluster, quasi and pseudo RCTs) evaluating CRP POCT in adult populations; qualitative studies investigating the experience, barriers, or facilitators to CRP POCT use; economic or cost-effective evaluations of CRP POCT; and mixed-methods and observational studies investigating CRP POCT use in adult or clinician populations. Studies focused on patient populations were restricted to adults (18 years or older) presenting with RTIs in primary care settings. Primary care settings included general practice and family practice settings. Patient population studies were excluded if the included data could not be separated from pediatric populations or non-RTI conditions. Systematic reviews, diagnostic accuracy studies, and protocols without outcome data were also excluded. Only papers published in English were included.

### 4.4. Screening

Search results were imported from their respective databases into Covidence and screened in a three-step process. After removing duplicates, two reviewers (N.M. and S.M.) independently screened titles, abstracts and full texts against eligibility criteria. Discrepancies were resolved by consensus, with a third reviewer (J.M.) as needed. Studies lacking sufficient CRP POCT outcome data were also excluded at full review with consensus.

### 4.5. Critical Appraisal

Although the Grading of Recommendations Assessment, Development and Evaluation (GRADE) approach was prespecified in PROSPERO (registration number CRD420251243866), it was not applied due to the predominance of non-randomised studies, which would have limited meaningful differentiation of evidence certainty [68]. Risk of bias was instead assessed using the Mixed Methods Appraisal Tool (MMAT) (Table S2–S5) [69] as it was developed and validated for the purpose of mixed-methods systematic reviews [70]. Data were extracted from each study into a standardised MMAT template in Microsoft Excel by two separate reviewers (N.M. and C.C.), with any disparities discussed and resolved. Most studies could be assessed accurately by the MMAT criteria; however, in the RCT appraisal, the blinding of interventions MMAT criterion could not be met in several studies, as it was irrelevant due to the open-label design of some RCTs. Further, in the appraisal category, common weaknesses were observed in the reporting and level of adherence to the intervention in trials, along with the reporting of randomisation techniques, inhibiting further assessment. Only two studies were deemed to be of low methodological quality, and both were in the mixed-methods category. Both studies lacked adequate methodological rationale for their design and outputs and therefore did not meet the respective MMAT criteria.

In summary, the included studies generally demonstrated moderate-to-high methodological quality. Most studies (65.7%; 23/35) met 75–100% of the criteria on their respective appraisal checklist and were categorised as high quality, while 28.6% (10/35) met 50–75% of the criteria and were classified as moderate quality. Only two studies 5.75% met fewer than 50% of the criteria and were therefore categorised as low quality. However, all studies were included in the review, as the MMAT appraisal tool discourages exclusion of studies with low methodological scores [69], so risk of bias was considered in the interpretation of results.

### 4.6. Data Extraction and Synthesis

Data were extracted in Microsoft Excel using a created template based on the five domains of the RE-AIM framework (Reach, Effectiveness, Adoption, Implementation and Maintenance) [21]. Individual indicators for each domain were iteratively developed and adapted from RE-AIM materials (https://re-aim.org/) and designed in reference to Glasgow, R. E. and P. E. Estabrooks [71] key domain questions for the context of this systematic review. Both quantitative and qualitative descriptive evidence served as data indicators for the five domains, with indicators summarised in Table 2.

Because this systematic review included different types of studies, not every study provided information for every domain. When information was missing, it was recorded as either not applicable or unavailable (N/A). Data extraction was performed by a single reviewer (N.M.) before being cross-checked by two reviewers (S.M., M.B.). Agreement was reached with 98.1% of the initial RE-AIM data extraction, with only a single extraction error identified during the verification process. Additional clarifications or details were incorporated where necessary, and any discrepancies or ambiguities were resolved through discussion and consensus among the team.

Following data extraction, the appropriate approach to data synthesis was determined. However, data pooling and individual data coding were also deemed inappropriate due to substantial heterogeneity across studies. Thus, to provide a comprehensive summary, RE-AIM domains were reported separately by study type, along with a narrative synthesis of key themes and findings across studies. Final interpretations were discussed and refined collaboratively among the authors.

## 5. Conclusions

Overall, the application of the RE-AIM framework identified key barriers to sustained and scalable implementation of CRP POCT in a primary care setting. Although gaps in reporting were noted across domains, this review found CRP POCT reduced prescribing, though usage rates varied significantly throughout the studies examined. Further, though limited long-term studies and sustainability assessments are available, the largest barriers to implementation, as viewed by clinicians, are related to operational concerns and financial reimbursement. Thus, further long-term sustainability research is required to address these structural and contextual barriers.

## Figures and Tables

**Table 1:** Summary descriptions of the included studies.

Authors/Date	Study Design	Location	CRP POCT Device	Participants and Sample Size	Primary Outcome or Aim	Comparison/ Intervention
Andreeva 2014 [22]	RCT	Russia	Afinion	101 adults with RTI from 4 practices	Antibiotic prescribing	CRP POCT vs. UC
Cals 2009a [23]	RCT	The Netherlands	Nycocard	431 adults with RTI from 20 practices	Antibiotic prescribing	CRP POCT, CT, Combination (CRP and CT) vs. UC
Cals 2013 [24]	RCT	The Netherlands	Nycocard	379 adults with RTI from 20 practices	Antibiotic prescribing	CRP POCT, CT, Combination (CRP and CT) vs. UC
Cals 2010 [25]	RCT	The Netherlands	Quikread	258 adults with RTI; from 11 practices	Antibiotic prescribing	CRP POCT vs. UC
Eley 2020 [26]	RCT	UK	Afinion	2297 adults with RTI from 16 practices; 134 adult patient surveys	Feasibility, acceptability and antibiotic prescribing	CRP POCT vs. UC
Jung 2024 [27]	RCT	France	QuikRead	342 adults with RTI (children excluded) from 26 practices	Antibiotic prescribing	CRP POCT vs. UC
Little 2013 [28]	RCT	UK, Belgium, The Netherlands, Poland, Spain	QuikRead	6771 adults with RTI from 246 international practices	Antibiotic prescribing	CRP POCT, CT, Combination (CRP and CT) vs. UC
Little 2019 [29]	RCT	UK, Belgium, The Netherlands, Poland, Spain	QuikRead	4830 adults with RTI from 168 practices	Antibiotic prescribing	CRP POCT, CT, Combination (CRP and CT) vs. UC
Llor 2024 [30]	RCT	Spain	Afinion	231 adults with RTI from 20 practices	Antibiotic prescribing	CRP POCT, CT, Combination (CRP and CT) vs. UC
Theodoropoulou 2025 [31]	RCT	UK	No device—Online questionnaire	769 adults from varying professions	Antibiotic prescribing expectations	CT, Combination (CRP and CT), vs. control
Anthierens 2015 [32]	Qualitative	UK, Belgium, The Netherlands, Poland, Spain	QuikRead	66 clinicians from 6 countries	Clinicians’ experiences with interventions	CRP POCT, CT, Combination (CRP and CT) vs. UC
Cals 2009b [33]	Qualitative	The Netherlands	Nycocard	20 clinicians	Clinicians’ experiences and attitude toward interventions	Combination (CRP and CT) vs. UC
Cals 2010 [34]	Qualitative	The Netherlands	Nycocard	20 clinicians from 10 practices,	Clinician experiences with CRP POCT	CRP POCT
Eley 2018 [35]	Qualitative	UK	Afinion	15 clinicians and 11 HCPs (11 interviews, 3 focus groups, 1 written response)	Participants’ experiences and attitude toward CRP POCT	CRP POCT (various uptake) vs. UC
Hardy 2017 [36]	Qualitative	US	No device	30 clinicians and HCPs from 5 practices	Clinicians view (barriers and facilitators) with CRP POCT	CRP POCT: Little to no experience with CRP POCT
Huddy 2016 [37]	Qualitative	Denmark, The Netherlands, Norway, Sweden, UK	No device	Stage 1: 8 clinicians experienced with CRP POCT; Stage 2: 10 multidisciplinary stakeholders	Stage 1: Clinicians’ experiences with CRP POCT Stage 2: barriers and facilitators to adoption	CRP POCT
Jung 2025 [38]	Qualitative	Germany	Rightsign	10 clinicians	Clinicians’ experiences and attitudes toward CRP POCT	CRP POCT
Moragas 2025 [39]	Qualitative	Spain	No device- prospective	18 HCPs	Prospective views on POCT	POCT
Rutter 2024 [40]	Qualitative	UK	FebriDx	12 adults with RTI (children excluded) and 10 HCPs from 6 practices	Views on CRP POCT	CRP POCT
Wood 2011 [41]	Qualitative	Belgium, Hungary, Poland, The Netherlands, Norway, Spain, Italy, UK	No device—prospective	80 clinicians and 121 adults with RTI from 9 primary care networks	Prospective views on CRP POCT (current views on use from Norway)	CRP POCT
Cals 2011 [42]	Economic	The Netherlands	Nycocard	Estimated adults with RTI from [23] population	Economic evaluation of CRP POCT	CRP POCT, CT, Combination vs. UC
Dick 2021 [43]	Economic	US	FebriDx	Estimated national level of adults with RTI in US outpatient settings	Economic evaluation of CRP POCT	CRP POCT vs. UC
Fawsitt 2022 [44]	Economic	Ireland	Not stated	National consultation rate data and literature were used to estimate adults with RTIs	Economic evaluation of CRP POCT	CRP POCT (with and without CT) vs. UC
Holmes 2018 [45]	Economic	UK	Afinion	Estimated number of adults with RTIs based on [56] population, supplemented with published data	Economic evaluation of CRP POCT	CRP POCT vs. UC
Hunter 2015 [46]	Economic	UK	Afinion	100 hypothetical estimates of adults with RTIs	Economic evaluation of CRP POCT	CRP POCT (GP + CRP, Nurse + CRP, GP + CRP + CT
Lubell 2018 [47]	Economic	Vietnam	Nycocard	Adults with RTI; population data linked to Vietnamese trial [57]	Economic evaluation of CRP POCT	CRP POCT vs. UC
Oppong 2013 [48]	Economic	Norway, Sweden	Not stated	370 adults with RTI	Economic evaluation of CRP POCT	CRP POCT vs. UC
Jakobsen 2010 [49]	Prospective observational	Norway, Sweden, UK	Quikread, Nycocard and No device	803 adults with RTI	Antibiotic prescribing	CRP POCT taken vs. not taken vs. UK subgroup (usual care)
Lindstrom 2015 [50]	Prospective observational	Sweden	Not stated	42 clinicians	Impact of CRP POCT on antibiotic prescribing decisions	CRP POCT
Markwart 2025 [51]	Prospective observational	Germany	Rightsign	1740 patient cases (RTI focus) from 49 practices	Utilisation, clinical impact and usefulness of CRP POCT in routine primary care	CRP POCT
Minnaard 2016 [52]	Prospective observational	The Netherlands	Afinion	939 adults with RTI recruited by 40 clinicians	Antibiotic prescribing decisions and guideline adherence	CRP POCT
Dixon 2022 [53]	Mixed methods	England (UK)	Afinion	12 clinicians and 4 allied HCPs	Antibiotic prescribing decisions	CRP POCT
Johnson 2018 [54]	Mixed methods	UK	Not stated	682 adults with RTI from 5 practices, 7 clinicians and commissioners’ response to survey	Implementation of CRP POCT: funding, barriers, enablers and policy incentives;	CRP POCT
Martinez-Gonzalez 2022 [55]	Mixed methods	Switzerland	No device- Web based survey	188 clinicians	Antibiotic prescribing decisions, knowledge, attitudes and barriers/facilitators	CRP POCT data
Hughes 2016 [56]	Mixed methods	UK	Afinion	94 adults with RTI using CRP POCT; 5 patients focus group participants and 11 survey responses from clinicians and staff	Antibiotic prescribing decisions, and clinician and patient experiences	CRP POCT

Abbreviations: POCT = point-of-care testing; UC = usual care; CT = communication training; GP = general practitioner; HCP = healthcare professional.

**Table 2:** RE-AIM framework domains and indicators used for data collection as developed from RE-AIM materials (https://re-aim.org/) and reference to the domain questions provided in Glasgow and Estabrooks [71].

RE-AIM Domain	Indicators for the RE-AIM Domain	Description of Indicators
Reach	Inclusion/Exclusion criteria	Inclusion of description of characteristics that determined eligibility of potential participants
	Sample size	Number of participants
	Characteristics of participants/non-participants	Inclusion and reporting of characteristics (e.g., age and sex) of eligible and ineligible participants
	Representativeness	Reporting and comparison of characteristics (regional/ethnicity and education information) of eligible population compared to target population
Effectiveness	Primary outcome	Reporting of main study outcome—commonly antibiotic reduction or change in decision making
	Negative outcomes	Reporting and assessment of negative or unanticipated consequences from intervention or during study
	Other study outcomes	Reporting of secondary or other outcome measures beyond primary outcome
	Qualitative perspectives	Inclusion of views of usefulness from clinicians and/or other healthcare professionals
Adoption	Uptake/Use rates	Inclusion and reporting of rates of intervention use
	Variations in use	Inclusion and reporting on how the intervention was used and by whom, e.g., clinicians vs. practice nurses
	Training/Compliance	Inclusion of reports on how participants were trained and complied with training
	Setting	Characteristics of location or setting type; e.g., within a practice, hypothetical scenario or discussion scenario
	Qualitative perspectives	Inclusion of patient and/or clinician perspectives on test engagement and acceptability
Implementation	Cost effectiveness/Reported costs	Description and determination of projected cost of intervention implementation
	Adherence/consistency	Reporting or description of non-adherence to guidelines or use beyond purpose
	Adaptations made	Reporting of adaptations to protocols or use
	Qualitative perspectives	Inclusion of views on workflow and barriers during study or trials
Maintenance	Follow-up studies	Reporting of long-term intervention outcomes (6 months or longer)
	Qualitative perspectives	Inclusion of views on future willingness, barriers and facilitators to further implementation

## Data Availability

All data generated or analysed during this study are included in this published article and its Supplementary Information File or upon request from the author.

## Supplementary Materials

The following supporting information can be downloaded at: https://www.mdpi.com/article/10.3390/antibiotics15090848/s1, Table S1: Search terms and databases searched; Table S2: Quality appraisal of quantitative studies (randomised controlled trials) [69]; Table S3: Quality Appraisal of qualitative studies [69]; Table S4: Quality appraisal of quantitative descriptive studies (economic) [69]; Table S5: Quality appraisal of mixed methods studies [69].

## Abbreviations

The following abbreviations are used in this manuscript:

AMR	Antimicrobial resistance
AMS	Antimicrobial stewardship
CRP	C-reactive protein
POCT	Point-of-care test
RCT	Randomised controlled trial
RE-AIM	Reach, effectiveness, adoption, implementation, maintenance
RTI	Respiratory tract infection
