# Supplementary material for: Implementation of C-Reactive Protein Point-of-Care Testing for Respiratory Tract Infections in Primary Care: A Systematic Review Using the RE-AIM Framework
Source: Antibiotics (Basel). 2026 Aug 31;15(9):848. doi: 10.3390/antibiotics15090848 (PMC13603815; doi:10.3390/antibiotics15090848)
Supplement: Supplementary file 1 [file antibiotics-15-00848-s001.zip › antibiotics-4465011-supplementary.pdf]

Supplementary Materials

Table S1. Search terms and databases searched.

Database Searched	Search Terms
**PubMed**	((("point of care test*" OR "point-of-care test*" OR "point of care diagnostic test*" OR "point-of-care diagnostic test*" OR "rapid diagnostic test*" OR "rapid diagnostics test*")) AND ("C-reactive protein" OR "C reactive protein" OR CRP) AND ("respiratory tract infection*" OR "respiratory infection*"))
**Scopus**	TITLE-ABS-KEY (("point of care test*" OR "point-of-care test*" OR "point of care diagnostic test*" OR "point-of-care diagnostic test*" OR "rapid diagnostic test*" OR "rapid diagnostics test*") AND ("C-reactive protein" OR "C reactive protein" OR CRP) AND ("respiratory tract infection*" OR "respiratory infection*"))
**EBSCO (MEDLINE, CINHAL)**	(("point of care test*" OR "point-of-care test*" OR "point of care diagnostic test*" OR "point-of-care diagnostic test*" OR "rapid diagnostic test*") AND ("C-reactive protein" OR "C reactive protein" OR CRP) AND ("respiratory tract infection*" OR "respiratory infection*"))
**ProQuest**	(("point of care test*" OR "point-of-care test*" OR "point of care diagnostic test*" OR "point-of-care diagnostic test*" OR "rapid diagnostic test*") AND ("C-reactive protein" OR "C reactive protein" OR CRP) AND ("respiratory tract infection*" OR "respiratory infection*"))
**CENTRAL**	#1: point of care test* OR point-of-care test* OR point of care diagnostic test* OR point-of-care diagnostic test* OR rapid diagnostic test* OR rapid diagnostics test*<br>#2: C-reactive protein OR C reactive protein OR CRP<br>#3: respiratory tract infection* OR respiratory infection*<br>#4: #1 AND #2 AND #3

Table S2. Quality appraisal of quantitative studies (randomised controlled trials) (Hong et al., 2018).

**MMAT Criteria**	***Paper (First Author and Year)***									
	Andreeva 2014	***Cals 2009a***	***Cals 2013***	***Cals 2010a***	***Eley 2020***	***Jung 2024***	***Little 2013***	***Little 2019***	***Llor 2024***	***Theodoropoulou 2025***
Clear research question(s)	1	1	1	1	1	1	1	1	1	1
Collected data allow to address the research question(s)	1	1	1	1	1	1	1	1	1	1
Randomization is appropriately performed	-	-	-	1	-	1	1	1	1	-
The groups comparable at baseline	1	1	1	1	-	1	1	1	0	1
There is complete outcome data	0	1	1	1	-	1	1	1	0	1
Outcome assessors blinded to the intervention provided	0	-	-	N/A*	N/A*	N/A*	N/A*	N/A*	0	0
Participants adhere to the assigned intervention	1	0	-	1	1	-	1	0	1	1
**MMAT Rating**	Moderate	Moderate	Moderate	High	Moderate	High	High	High	Moderate	Moderate

Key: 1 = yes, 0 = no, - = can’t tell, N/A* Open-label RCT does not apply.

Table S3. Quality Appraisal of qualitative studies (Hong et al., 2018).

MMAT criteria	*Paper (First Author and Year)*									
	Anthierens 2015	*Cals 2009b*	*Cals 2010b*	*Eley 2018*	*Hardy 2017*	*Huddy 2016*	*Jung 2025*	*Moragas 2025*	*Rutter 2024*	*Wood 2011*
Clear research question(s)	1	1	1	1	1	1	1	1	1	1
Collected data allow to address the research question(s)	1	1	1	1	1	1	1	1	1	1
Qualitative approach is appropriate for research question	1	1	1	1	1	1	1	1	1	1
Qualitative collection methods are appropriate	1	1	1	1	1	1	1	1	1	1
Findings adequately derived from data	1	1	1	1	-	1	1	1	1	1
Interpretation of results sufficiently substantiated by data	1	1	1	1	1	1	1	1	1	1
Coherence between qualitative data sources, collection, analysis and interpretation	1	1	1	1	1	1	1	1	1	1
**MMAT Rating**	High	High	High	High	High	High	High	High	High	High

Key: 1 = yes, 0 = no, - = can't tell.

Table S4. Quality appraisal of quantitative descriptive studies (economic) (Hong et al., 2018).

MMAT Criteria	*Paper (First Author and Year)*						
	Cals 2011	*Dick 2021*	*Fawsitt 2022*	*Holmes 2018*	*Hunter 2015*	*Lubell 2024*	*Oppong 2013*
Clear research question(s)	1	1	1	1	1	1	1
Collected data allow to address the research question(s)	1	1	1	1	-	1	1
Sampling strategy is relevant to address the research question(s)	1	1	1	0	-	-	1
Sample is representative of target population	1	1	1	0	-	-	1
Measurements are appropriate	1	1	1	1	1	1	1
The risk of nonresponse bias is low	1	-	1	1	1	1	1
The statistical analysis is appropriate to answer the research question	1	-	1	1	1	1	1
**MMAT Rating**	High	Moderate	High	Moderate	Moderate	Moderate	High

Key: 1 = yes, 0 = no, - = can't tell.

Table S5. Quality appraisal of mixed methods studies (Hong et al., 2018).

Criteria type	MMAT Criteria	*Paper (First Author and Year)*							
		Jakob-sen 2010	***Lindstrom 2015***	***Markwart 2025***	***Minnaard 2016***	***Dixon 2022***	***Johnson 2018***	***Martinez-gon-zalezGonzalez 2022***	***Hughes 2016***
Screening Questions	Clear research question(s)	1	1	1	1	1	1	1	0
	Collected data allow to ad-dress the research ques-tion(s)	1	1	1	1	1	1	1	1
Quantitative non RCT	Participants are representa-tive of the target popula-tion	1	1	1	1				
	Measurements are appro-priate regarding both the outcome and intervention (exposure)	1	1	1	1				
	Complete outcome data	1	1	1	1				
	Confounders accounted for in design and analysis	1	1	0	1				
	During study period, inter-vention is administered as intended	1	1	1	1				
Mixed meth-ods	Adequate rationale for us-ing a mixed methods de-sign to address research question					1	0	1	0
	Different components are effectively integrated to answer research question					1	1	1	1
	Outputs of integration of qualitative and quantitative components are adequately interpreted					1	0	1	0
	Divergencies and incon-sistencies between quanti-tative and qualitative re-sults are adequately ad-dressed					1	-	1	1
	Different components of the study adhere to quality criteria of each tradition of methods					1	-	1	0
**MMAT Rating**		High	High	High	High	High	Low	High	Low

Key: 1 = yes, 0 = no, - = can't tell.

References

69. Hong, Q.; Pluye, P.; Fàbregues, S.; Bartlett, G.; Boardman, F.; Cargo, M.; Dagenais, P.; Gagnon, M.-P.; Griffiths, F.; Nicolau, B.; et al. Mixed Methods Appraisal Tool (MMAT), Version 2018; Registration of Copyright (#1148552); Canadian Intellectual Property Office, Industry Canada: Gatineau, QC, Canada, 2018. Available online: https://www.nccmt.ca/knowledge-repositories/search/232 (accessed on 20 December 2025).
